# Progress of stem/progenitor cell-based therapy for retinal degeneration

**DOI:** 10.1186/s12967-017-1183-y

**Published:** 2017-05-10

**Authors:** Zhimin Tang, Yi Zhang, Yuyao Wang, Dandan Zhang, Bingqiao Shen, Min Luo, Ping Gu

**Affiliations:** Department of Ophthalmology, Ninth People’s Hospital, Shanghai Jiao Tong University School of Medicine, Shanghai, 200011 People’s Republic of China

**Keywords:** Retinal degeneration, Stem/progenitor cells, Clinical trials, Proliferation, Differentiation, Transplantation

## Abstract

Retinal degeneration (RD), such as age-related macular degeneration (AMD) and retinitis pigmentosa, is one of the leading causes of blindness. Presently, no satisfactory therapeutic options are available for these diseases principally because the retina and retinal pigmented epithelium (RPE) do not regenerate, although wet AMD can be prevented from further progression by anti-vascular endothelial growth factor therapy. Nevertheless, stem/progenitor cell approaches exhibit enormous potential for RD treatment using strategies mainly aimed at the rescue and replacement of photoreceptors and RPE. The sources of stem/progenitor cells are classified into two broad categories in this review, which are (1) ocular-derived progenitor cells, such as retinal progenitor cells, and (2) non-ocular-derived stem cells, including embryonic stem cells, induced pluripotent stem cells, and mesenchymal stromal cells. Here, we discuss in detail the progress in the study of four predominant stem/progenitor cell types used in animal models of RD. A short overview of clinical trials involving the stem/progenitor cells is also presented. Currently, stem/progenitor cell therapies for RD still have some drawbacks such as inhibited proliferation and/or differentiation in vitro (with the exception of the RPE) and limited long-term survival and function of grafts in vivo. Despite these challenges, stem/progenitor cells represent the most promising strategy for RD treatment in the near future.

## Background

Human eye formation involves a complex process requiring elaborate epithelial movements and cellular choreography. The retinal pigmented epithelium (RPE), Bruch’s membrane/choroid and retinal photoreceptor cells are the dominant cell types participating in light-perception. Any progressive degeneration of these cells can lead to retinal degeneration (RD). RD includes diverse ocular diseases, namely, age-related macular degeneration (AMD), retinitis pigmentosa (RP), diabetic retinopathy and glaucoma. In particular, the main characteristic of AMD (mainly affecting elderly individuals worldwide) is an abnormal decrease of RPE leading to secondary photoreceptor dysfunction, while RP (the leading cause of irreversible blindness in paediatric and young populations) is a hereditary retinal degenerative disease characterized by the progressive death of photoreceptors [1]. Although AMD and RP differ in pathological progress, they impinge upon a common final pathway of photoreceptor loss. It is evident that the prevalence of blindness due to RD is increasing according to the latest systematic analysis of causes of blindness around the world from 1990 and 2010, and the prevalence linked to macular degeneration was 5 and 7%, respectively [2]. Blindness is associated with devastating impacts on functional abilities and quality of life, leading to increased health care resource utilization and higher patient support cost [3]. In addition, the degree of vision impairment for different individuals varies depending on age, disease stage, and occurrence time emphasizing the need for new proposals to help prevent or reverse RD. A number of related strategies have been explored, such as neurotrophic factor supports, electronic retinal prostheses and pharmacological treatments (e.g., anti-vascular endothelial growth factor therapy used in wet AMD treatment [4]). However, the current strategies only retard the progression of RD, which is not yet curable. The regrowth of retinal cells is still limited although Joel Schuman et al. have shown neurogenesis in the adult mice retina [5]. Stem/progenitor cell-based therapy could play a critical role in sight restoration by replacing missing retinal cells and/or rescuing remaining cells. Here, different stem/progenitor cells can be obtained from two broad lines. (1) Ocular-derived progenitor cells, e.g., retinal progenitor cells (RPCs), are located in the inner layer of the optic cup where nearly all retinal cell types initially differentiate from [6]. It has been reported that foetal and postnatal-derived RPCs could express immature markers, indicative of a retinal stem-cell state [7, 8]. (2) Non-ocular-derived stem cells (with the potential to self-renew and produce different cells including RPE, photoreceptors, etc.), include embryonic stem cells (ESCs) [9], induced pluripotent stem cells (iPSCs) [10], mesenchymal stromal cells (MSCs) (particularly bone marrow mesenchymal stromal cells (BM-MSCs) [11] and adipose-derived stromal cells (ADSCs) [12]).

In this review, an in-depth analysis of RPCs, ESCs, iPSCs and MSCs was conducted. Identifying the main advantages and disadvantages of these cells is the key to selecting the most promising candidates that could be applied in RD treatment (Table 1). Among these four cell types, RPC populations used as allografts have shown immune privilege and a relatively simple manufacturing process [13], and they are one of the best options. However, the challenge of acquiring adequate progeny still remains. ESCs and iPSCs have the potential to replace retinal cells. However, the vital issues in the use of ESCs and iPSCs are ethical and biosafety concerns (like genetic abnormalities [14]). In contrast, MSCs mainly provide trophic support to slow down retinal cell degeneration instead of replacing the missing retinal cells. At present, clinical trials are underway to evaluate three major issues (Table 2): (1) safety, (2) efficacy, and (3) efficiency. Based on the ability of transplanted cells to differentiate and replace the missing photoreceptors or simply protect the remaining photoreceptors during degenerative process, cell-based therapy appears to be valid so far [15–17]. Stem/progenitor cells present challenges related to their proliferation and/or differentiation into target cells in vitro, but that does not apply to RPE [18]. Other factors to consider are limited likelihood of long-term graft survival and host functional restoration in vivo. Even then, it is anticipated that this will progressively become a promising method for visual restoration in the near future because of the concerted research efforts worldwide.

**Table 1:** Comparison of four types of stem/progenitor cells for RD clinical application

Cell types	RPCs	ESCs	iPSCs	MSCs
Derivation/generation sources	Foetal and postnatal retina	Developing embryos	Terminally differentiated tissues	Developmentally mature organs
Advantages	Simplicity, accessibility and safety (minimal trauma); immune privilege; ready neuroprotection; no tumourigenicity; no requirement of immunosuppressive drugs	Differentiation into various retinal cell types; providing abundant donor cells	Without ethical concerns; low risk of immune rejection (autologous hiPSC derivatives); gene therapy	Trophic support; immunosuppression
Disadvantages	Low rate of cell proliferation	Ethical concerns; tumourigenicity; requirement of immunosuppressive treatment throughout life	Low differentiation efficiency; biosafety concerns (e.g., genetic abnormalities)	Low rate of cell migration and differentiation

**Table 2:** Clinical trials using stem/progenitor cell-based therapeutics in RD

Cell therapy	Identifier	Phase	Institution	Location	Principal investigator	Study start date	Study status	Diseases and enrolment	Single dose	Months of follow-up	Intervention	Safety issues	Visual acuity and number of patients			Other trials
													Improved	Stable	Decreased	
hRPCs	NCT02320812	I/IIa	jCyte	California, US	Henry Klassen	June 2015	Ongoing but not enrolling patients	28 RP	500,000 –3,000,000 cells	12	Intravitreal	No data	No data	No data	No data	ReNeuron (Boston: Phase I/II, 15 RP)
ESCs → RPE	NCT01345006	I/II	Advanced Cell Technologies	California, US	Steven Schwartz	April 2011	Completed	9 SMD	50,000–150,000 cells	22	Subretinal	None	10	7	1	Pfizer [London: Phase I, 10 AMD (wet)]
	NCT01344993							9 AMD (dry)								
	NCT01625559	I	CHABiotech	Seoul, South Korea	Won Kyung Song	September 2012	Unknown	2 SMD	50,000 cells	12	Subretinal	None	3	1	None	
	NCT01674829	I/IIa						2 AMD (dry)								
	NCT02286089	I/II	Cell Cure Neuroscience	Jerusalem, Israel	Ytzhak Hemo	April 2015	Enrolling patients	15 AMD (dry)	50,000–500,000 cells	12	Subretinal	No data	No data	No data	No data	
iPSCs → RPE	UMIN000011929	I	RIKEN	Kobe, Japan	Masayo Takahashi	September 2014	Suspended	1 AMD (wet)	1.3 mm × 3 mm RPE sheet	12	Subretinal	None	None	1	None	National Eye Institute (Preclinical, AMD)
BM-MSCs	NCT01068561	I	University of Sao Paulo	São Pauloin, Brazil	Rubens C Siqueira	May 2009	Completed	3 RP and 2 cone-rod dystrophy	10,000,000 cells	10	Intravitreal	None	4	1	None	Red de Terapia Celular (Spain: Phase I, 10 RP); Al-Azhar University [Egypt: Phase I/II, 1 AMD (dry)]
	NCT01560715	II				January 2011	Completed	20 RP		12			20 (transitorily)	None	None	
BM CD34+ cells	NCT01736059	I	University of California, Davis	California, US	Susanna S Park	July 2012	Enrolling patients	6 RD or ischaemic disorders	3,400,000 cells	6	Intravitreal	None	6	None	None	

### Progress in the study of stem/progenitor cells in RD

We focused on advances in two broad categories of stem/progenitor cells, i.e., ocular-derived progenitor cells and non-ocular-derived stem cells, which were studied broadly in various animal models of RD (mouse, rat, rabbit, pig, monkey, etc.) and applied in clinical trials.

### Ocular-derived progenitor cells

As one kind of ocular-derived progenitor cells, the multipotent RPCs are specially mentioned. Based on some research over the past a few years, RPCs displaying stem cell properties hold hope for RD treatment [19].

### RPCs

#### Progress in the study of non-human RPCs

**Fig. 1:**
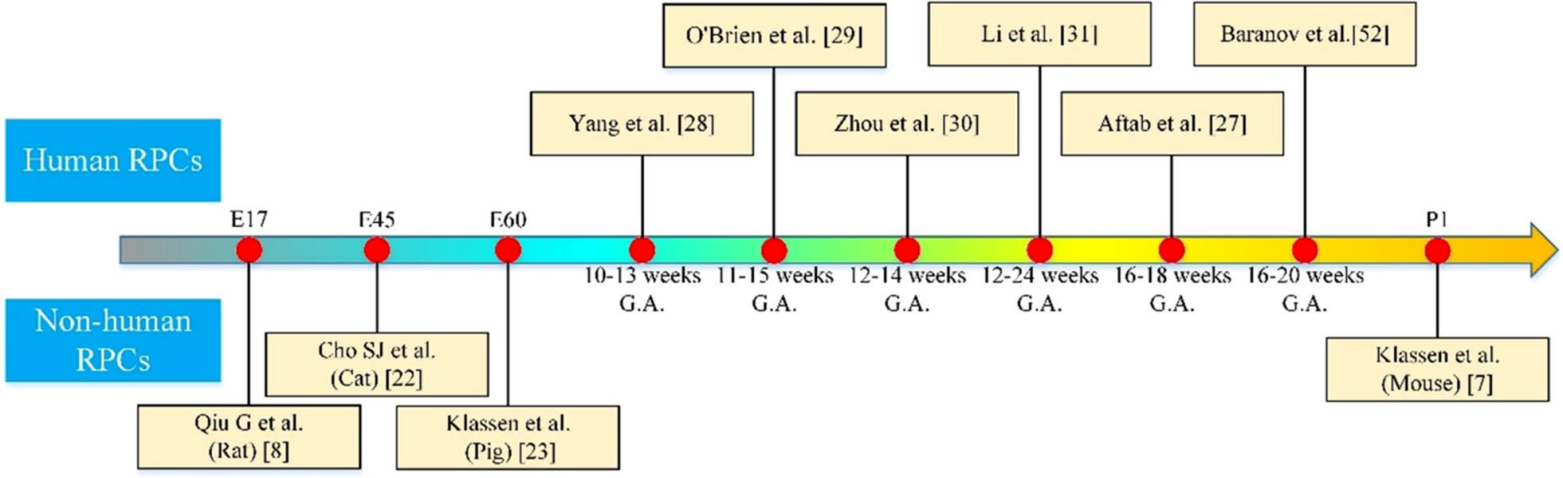
The period for isolating and culturing RPCs. *RPCs* retinal progenitor cells, *E* embryonic day, *P* postnatal day, *G.A.* gestational ages

It was observed that RPCs isolated and cultured from different gestational or postnatal periods (Fig. 1) could differentiate into various retinal cell types at different times (e.g., Müller glial cells, rod photoreceptors and bipolar neurons [8, 20]). In the work of Klassen et al., the host was a rho−/− mature mouse that experienced light-mediated behaviour improvement resulting from the transplantation of RPCs from postnatal day one green fluorescent protein (GFP)-transgenic mice, and the grafted RPCs that showed photoreceptor rescue in the outer nuclear layer (ONL) and were integrated widely into the inner retina [7]. The RPCs of embryonic day 17 rat grown in serum-free defined media using all-trans retinoic acid (RA) demonstrated down regulation of nestin (a critical retinal progenitor-related marker) and co-expression of extensive mature retinal specific markers including rhodopsin, protein kinase C-α, cellular retinaldehyde binding protein and neuro-filament 200 [8]. This suggested that RPCs at early embryonic period also maintain their self-renewing and multilineage potential. In the transplantation experiments, many scientists have focused on mouse [7] or rat [21] to confirm the potential of RPCs for replacing damaged cells in RD; thus, large animal models, such as cat [22] and pig [23], are in demand to continue the work with RPCs. Cat retinal sheets at gestational day 42 containing undifferentiated RPCs were transplanted subretinally into the eyes of four dystrophic Abyssinian cats with progressive rod-cone degeneration, and the retinas of two hosts showed good integration of the transplants and lamination of photoreceptors without immunoreactivity [24]. Transplantation studies in pig also have great significance for biomedical applications because the pig eye is similar to the human eye in physiology, anatomy and metabolism [25, 26]. Donor cells isolated from foetal GFP-transgenic pigs were successfully implanted into pigs with RD, which resulted in sufficiently long-term survival of grafted cells to populate the injured areas and exhibit morphologic differentiation without exogenous immune rejection [13]. The outcomes were similar to those observations of RPCs that were obtained from mouse xenografts [7]. In general, these findings contribute to speculation on the utilization of human retinal progenitor cells (hRPCs).

#### Progress in the study of hRPCs

Theoretically, hRPCs could also be used for treatment of RD through transplantation. For example, it is possible to dissociate foetal and postnatal-derived hRPCs so that photoreceptors are generated to integrate into the recipient’s retina (Fig. 1). Aftab et al. isolated hRPCs from donor tissue at 16–18 weeks gestational age, which proliferated for at least six passages in vitro, and some of these hRPCs expressed rhodopsin and integrated within the retina of rho(−/−) mice [27]. Yang et al. [28] found that human retina collected between gestational weeks 10 and 13 could produce progenitors that expanded in vitro for multiple generations (up to passage eight). Some research suggests that the best donors RPCs are isolated from 11 to 15 weeks gestational age when neurons begin to mature into photoreceptors and after mitosis has ceased [29], indicating the importance of selecting the correct gestation period to isolate and culture hRPCs. However, for the purpose of discovering the best donors of RPCs as a treatment strategy for RD, the stages at which hRPCs could survive long enough ex vivo and yield maximum the number of target cells still need to be determined. Following transplantation into the subretinal space (SRS) of the Royal College of Surgeons (RCS) rats, the RPCs obtained from human foetal retina during the 12th to 14th week of gestation self-renewed and differentiated into specialized retinal cells for at least three months without forming tumours [30]. Partial prevention of the deterioration of visual acuity was also achieved by grafting RPCs from human foetal (16 weeks) neuroretina into RCS rats [1]. Li et al. transplanted human foetal RPCs (12–24 weeks) into mini-pigs with light-induced RD and found that subretinal transplantation was successful in 15/25 eyes (60%), and the host animals showed visual functional improvement without graft rejection over 12 months [31]. There is a common misconception that ciliary epithelium (which can differentiate into rod photoreceptors, bipolar neurons and glial cells [32]), and Muller glia (which can de-differentiate into RPCs [33]) are the main cells with stem cell characteristics in adult human eyes. In fact, adult human eyes contain RPCs [28] similar to those isolated from rodent eyes [34]. Recently, adult hRPCs and human activated microglia in co-culture were investigated to assess proliferation and expression of the photoreceptor marker recoverin [35]. Regardless of whether RPCs are obtained from rodents, non-rodent animals or humans, they can commit to RPE or photoreceptor fates.

#### The main advantages and disadvantages of RPCs

The main issue facing RPC studies is how to obtain sufficient donor cells for transplantation studies. Even though treatment of the diseased macula alone rather than the entire retina may suffice, the efficiency of RPC differentiation and integration should be taken into consideration as well. Notably, some efficient protocols discussed below have been developed: (1) supplementation with other defined factors (such as ciliary neurotrophic factor [36] and insulin-like growth factor-1 [37]), which promotes differentiation into retinal specific cells within a shorter period compared with traditional growth factors [38]; (2) manipulation of microRNAs (22-nucleotide single-non-coding RNAs) [39–41], e.g., the lethal-7 family [42]), which excellently mimicks the natural production of retinal cells; and (3) retinal tissue engineering for the survival and differentiation of RPCs using poly(l-lactic acid) and poly(lactic-co-glycolic acid) polymer [43], electrospun nanofibrous membrane employed in our laboratory [44, 45], and hyaluronan and methylcellulose designed by Ballios [46]. Specifically, the delivery systems for RPCs or other specific differentiated cell types may be one of the most promising approaches for treating late-stage RD because of their overwhelming benefits. Examples of such benefits include the following: (1) the integration and survival rate of implanted RPCs could be enhanced greatly compared to conventional bolus injections that may result in massive efflux of new cells and cell death; (2) some of these approaches have received US FDA approval for clinical applications, e.g., poly(ε-caprolactone) [47]; (3) some of these approaches have high biocompatibility of nanofibrous structures, are non-toxic and exhibit good mechanical properties. Before the use of such biomaterials, their mechanical, biological and degradable properties will require further testing based on the findings of more basic research.

#### Clinical studies of RPCs

The employment of RPCs combined with defined factors, microRNAs manipulation, and retinal tissue engineering illustrates that RPCs have enormous potential for clinical application. Early clinical studies of allogeneic human foetal neuro-retinal cells that were transplanted in 14 patients with RP were performed by Das et al. [48, 49]. Several months later, new visual sensation was regained, and no detrimental effects of the grafts were reported. Based on such previous favourable outcomes, in June 2015, Klassen et al. conducted the first Phase I/IIa trial (NCT02320812) using foetal tissue-derived RPCs after receiving authorization from the FDA. The cells were delivered in suspension as an intravitreal injection into patients with RP [50]. The official primary outcome of this study will be published in July 2017. Similarly, the FDA approved another clinical test of RPCs initiated in Boston using a subretinal method in advanced RP individuals [51]. One merit of these transplantations was that RPCs obtained from human retina showed unprecedented immune privilege and simplicity, which could help scientists to determine the donor cells with maximum potential to be applied in RD treatment. The major problem is to obtain an adequate source of donor cells for effective transplantation. Currently, Baranov et al. have reported that hRPCs from 16 to 20 weeks gestational age in low oxygen conditions (3% O_2_) could be passaged a maximum of 16 times [52]. It is possible that with time, RPCs may proliferate for 20–30 passages or more in the near future, pending improvements to current techniques.

### Non-ocular-derived stem cells in RD

In this part, three main non-ocular-derived stem cells, ESCs, iPSCs and MSCs, are described in detail.

### ESCs

#### Progress in the study of non-human ESCs

In recent years, there has been a push to direct retinal cells differentiation from ESCs since their isolation from mouse by Martin et al. [53]. The mouse can serve as a perfect model for understanding the mechanisms regulating the specification of retinal neurons. A subset of neural progenitors derived from mouse ESCs showed an excellent ability to differentiate into the photoreceptor lineage in vitro [54]. To investigate the potential of these cells for transplantation. Meyer et al. [55] implanted GFP transgenic mouse ESC-derived neural precursors into the intravitreal space of mnd mice. The grafts were integrated into most layers of retinal tissue, and they exhibited typical properties of neurons through the expression of synaptic and neuronal markers such as NeuN and calretinin. To further evaluate the treatment potential of these cells for rats, mouse ESC-neural precursors were implanted subretinally into 20-day-old RCS rats with AMD [56]. The results showed incorporation of cells into layers of photoreceptor nuclei (up to eight rows) and improvement in the delayed photoreceptor degeneration, which could benefit the retinal structure and function. Compared with the feasible transplantation of rodent ESC derivatives, transplantation of primate ESCs also works. Takahashi et al. described a process for inducing retinal cells from monkey ESCs and demonstrated their properties in vitro and in vivo [57, 58]. By co-culturing ESCs with RPE and treating ESCs with RA in vitro, monkey ESC derivatives survived to organize into the tissue of nude mice and furthermore exhibited extensive rhodopsin expression, although they also formed teratomas [59].

#### Progress in the study of human embryonic stem cells (hESCs)

With an increasing understanding of the pluripotent nature of ESCs, a number of labs worldwide have established hESC derivation and culture protocols [60]. A milestone in this field was the work of Thomson, who isolated ESC lines from human blastocysts in 1998 [9]. Thereafter, Lamba et al. found that up to 80% of retinal progenitors derived from hESCs, comparable to those generated from human foetal retina, expressed a similar gene profile under appropriate culture conditions, and some hESC-progenitors with functional glutamate receptors under co-culture with retinas of degenerated mice were integrated into the retina and improved the expression of photoreceptor-specific markers [61]. Recently, Plaza Reyes et al. [62] established a rabbit model of geographic atrophy that allowed hESC-RPE transplantation with long-term integration and photoreceptor rescue capability. Despite the fact that hESC-derived retinal cells in rodents and large-eyed models could replace and rescue photoreceptors, the efficacy of the method still needs to be studied more thoroughly in primate models. In 2016, Shirai et al. grafted hESCs-retina into a newly developed monkey model, and the grafts differentiated into photoreceptors (rods and cones) that developed into structured ONL, and formed host-graft synaptic connections [63]. Accumulating evidence in hESC transplantation efficacy emanating from techniques development and various animal studies have shown that ESCs are considered to be suitable pluripotent cells for RD treatment.

#### The main advantages and disadvantages of ESCs

ESCs also present inherent challenges that create considerable boundaries to their effective use. For instance, ESCs exhibit the possibility of tumourigenicity. Chaudhry et al. induced ESCs into neural progenitors before transplantation into rd12 mice [64]. Six weeks after transplantation of both ESCs and neural progenitors, the robust ESCs more easily formed teratomas than the neural progenitors that proliferated at a slower rate. These observations suggest that transplantation of pre-induced ESCs may be a useful procedure that could reduce the risk of tumour formation. When hESC-RPE cells were implanted (in suspension or as a polarized monolayer on a parylene membrane) into immuno-deficient rats, the donors survived for at least 12 months with negligible teratomas [65]. Despite this study indicating similar favourable outcomes using human cells, the tumourigenic potential of ESCs cannot be ignored [64]. With the optimization of embryo culture, the pluripotent ESCs from inner cell mass can be induced to differentiate into almost any cell type in the body. However, these limitless possibilities of ESCs have also led to ethical concerns, and there are difficulties in limiting the differentiation of ESCs into ideal photoreceptor cells in vitro and in vivo [66]. As a result, protocols are being established to conform to Good Manufacturing Practices, which include (1) the regulation of micro-environment to obtain a relatively high yield of target cells [67]; (2) manipulation of signalling pathways (such as Rho-associated, coiled-coil protein kinase inhibitions) to help eye formation and enhance hESC expansion [68]; (3) feeder-free strategies to improve differentiation efficiency [69]; (4) xeno-free techniques (free of human or animal derivatives) to greatly reduce the risk of contamination in harvested cells and decrease immune response [70]; (5) three-dimensional retina cultures intended to form complete and organized retinas to better mediate retinal repair [71, 72]; 6) bioengineering techniques, such as porous honeycomb-like films [73] and ultrathin substrates [74] to ensure high adherence and differentiation of hESC-RPE before and after implantation.

#### Clinical studies of ESCs

Until now, sufficient preclinical models including mouse, rat, rabbit, cat [75], pig [76] and monkey have served as a strong base to replicate hESC-retinal cells. Those models share common mechanisms conserved in different species (including human beings), which could push this technique towards clinical trials. The first US FDA-approved clinical study involving subretinal transplantation of hESCs-RPE (NCT01345006 and NCT01344993) was conducted in 2011 by Schwartz et al., who implanted a low dose (50,000 cells) of RPE into two patients (Stargardt’s macular dystrophy (SMD) and non-exudative (dry) AMD). The visual acuity was improved by at most 5 letters in the SMD patient, and 12 letters in the dry AMD patient. Neither patient reported serious systemic or ocular adverse reactions during the postoperative period of four months [77]. In the follow-up trials, three dose cohorts (50,000, 100,000, and 150,000 cells) were transplanted into nine patients with SMD and nine with advanced dry AMD. Improvement of visual function was reported in ten treated eyes 22 months after transplantation [15]. In addition, the first Asian clinical trial involving four patients who were injected with 50,000 hESCs-RPE per eye was performed by Song et al. in 2015. No evidence of serious safety issues was observed, and 9–19 letters improvement of visual acuity were achieved in three patients after 12 months of follow-up [16]. More recently, imminent phase trials in some cities such as Jerusalem (Israel) are currently enrolling patients with AMD. Although ESCs can differentiate into various retinal cell types and provide millions of target cells required for transplantation, allografts of ESC-derived cells involve many unresolved issues such as ethical concerns, the potential for tumour formation and the requirement for lifelong immunosuppressive therapies.

### iPSCs

#### Progress in the study of non-human iPSCs

Application of iPSCs did not begin until the differentiation from “spontaneously” hESCs to retinal specific lineage was observed. Since then, advancements in this field have been rapid. iPSCs were converted from several types of somatic cells by introducing reprogramming factors. Yamanaka, the famous pioneer of these methods, induced mouse embryonic and adult fibroblasts into an embryonic-like state via viral transduction with the following four factors: Oct3/4, Sox2, c-Myc, and Klf4 [10, 78]. That iPSCs can re-differentiate into different retinal cell types and tissues morphologically and functionally by utilizing almost the same protocols of ESCs as above was later validated. Hirami et al. treated mouse iPSCs and human induced pluripotent stem cells (hiPSCs) with Wnt and Nodal antagonists in suspension culture, which promoted retinal differentiation and expression of RPE specific markers [79]. Mouse iPSCs are valuable as a cell source for RD treatment. Following transplantation into immune-compromised mice with RD, adult mouse iPSC-derived retinal progenitors were incorporated into ONL, which led to the improvement of electro-retinal function, although they later formed teratomas [14, 80]. Additionally, pig iPSC-derived rod photoreceptors can also integrate into the ONL of damaged pig eyes three weeks after subretinal transplantation, thus providing a foundation for future experiments using the pig as a model for stem/progenitor transplantation [81].

#### Progress in the study of hiPSCs

Buchholz et al. found that hiPSCs can also be isolated and cultured into functional RPE that were quantitatively similar to hESCs-RPE and human foetal RPE. The analysis of rod outer segment phagocytosis, gene and protein expression supported the finding that the differentiation potentials of hiPSCs and hESCs were similar [82, 83]. Hu et al. reported that some iPSCs lines showed remarkable variation in their efficiency even though they caused similar retinal induction [84]. The neuronal differentiation efficiency was significantly lower and variable among hiPSCs compared to hESCs, which indicated that the differentiation potency and capacity of iPSCs need to be improved [85]. The use of hiPSCs should be supported by preclinical testing that would determine whether they could restore visual function. In vivo, the visual function of blind Lrat(−/−) and Rpe65(−/−) mice was recovered after hiPSCs-RPE were transplanted subretinally to replace the dysfunctional RPE [86]. However, a treatment scheme using RPE alone may not be beneficial for all RD. Thus, photoreceptor replacement is also a necessary procedure [87]. When purified hiPSC-photoreceptors were transplanted into normal mice, the grafts took residence in the retina and expressed retina-specific markers [88]. Additionally, following implantation into mice with end-stage RD, the photoreceptors from iPSCs were connected to retinal neurons, thereby contributing to the improvement of visual function [89, 90]. However, compared with iPSC-derived RPE transplantation, there remain some unresolved technical difficulties with photoreceptors since more donor cells, higher functional efficacy in restoration of outer segments, and greater manipulation of culture environment are required. Most importantly, Stanzel et al., found that polarized human foetal and adult RPE as a monolayer could survive in rabbit SRS and maintain their near-native characteristics [91], which indicated that polarized RPE monolayer transplantation for RD treatment plays an important role in differentiation into neural retinal cells and protection of the retina due to their blood-ocular barrier function [65, 91, 92]. To some extent, the differences between them could help understand why there are more than six clinical trials with ESCs and iPSCs-derived RPE but not photoreceptors (Table 2). All these procedures in the use of iPSCs offer an opportunity to recapitulate the formation of human retinal cells in vitro and in vivo.

#### The main advantages and disadvantages of iPSCs

Whether disease-specific iPSCs can produce retinal cells and subsequently enable their use in autologous transplantation to avoid immune rejection is one of the distinctive differences between iPSCs and ESCs. Disease-specific hiPSCs under feeder- and serum-free adherent conditions supplemented with exogenous delivery of basic fibroblast growth factors, RA, and noggin exhibited specific molecular markers and similar RPE morphology [93]. In 2012, Li et al. initially developed a patient-specific iPSC-derived RPE transplantation protocol for direct functional recovery and additionally underpinned the feasibility of autologous transplantation [94]. Additional developments indicated that feasible autologous hiPSC derivative transplantation, especially using hiPSC-derived RPE [95], may be enhanced via gene repair [96, 97]. Laboratory-based discovery of intrinsic genetic networks in selected models has allowed the mapping of extrinsic signalling pathways that can be modulated by endogenous and exogenous factors. Recently, one breakthrough in the understanding of clustered regularly interspaced short palindromic repeats/Cas genome editing was achieved, and this technique may eventually address gene mutations in retinal heterogeneity such as RP [98, 99]. In general terms, iPSCs possess several notable differences from ESCs as follows: (1) a lower and more variable differentiation efficiency, (2) no ethical concerns, (3) a relatively low risk of immune rejection (for autologous iPSC-derived transplantation), and (4) feasible gene repair.

#### Clinical studies of iPSCs

The progress in iPSC replacement therapy is accelerating towards the clinic. Takahashi’s group (RIKEN in Kobe, Japan, September 2014) performed the pilot iPSC trial (UMIN000011929) of autologous iPSC-derived RPE sheets in a female suffering from exudative (wet-type) AMD [92]. The latest outcome, published on 16 March 2017, confirmed that her visual function had not improved or declined one year after surgery [100]. However, since three copy-number variants and three single-nucleotide variations in iPSCs were found in March 2015, scientists decided to suspend their original plans for applying for regulatory permission in Japan. After that, they moved their field into allogeneic transplantation by matching human leukocyte antigen and then enrolled the first Japanese male to receive allogeneic iPSC-RPE in suspension instead of sheets on 28 March 2017 [101, 102]. These reprogrammed iPSCs exhibited similar marker genes, morphology and growth properties of ESCs, but the lower differentiation potential and extra biosafety issues (especially tumourigenicity) required sufficient time to evaluate. Many scientists endeavoured to better address safety concerns via producing virus-free iPSCs [103–105]. Unfortunately, it is still unclear whether the reprogramming process of iPSCs integrated with these transcription factors causes iPSC abnormalities or oncogenesis of human hosts.

### MSCs

#### Progress in the study of BM-MSCs

A wide range of MSCs with multi-lineage differentiation originating from bone marrow, adipose tissue, umbilical cord, amniotic-fluid, etc., are considered as one therapeutic option for RD. Two main comparative sources of MSCs, BM-MSCs and ADSCs, were investigated to provide information for the study of retinopathies. For example, 35 days after transplantation into the rho(−/−) mouse, the mouse BM-MSCs exhibited neuronal and glial morphologies, incorporated into the host’s neuroretina layers and prolonged photoreceptor survival [11]. The BM-MSCs obtained from Pcrx2K-lacZ transgenic mice using mouse retinal cell culture in vitro were employed to delay photoreceptor apoptosis via factor secretion, and BM-MSCs injected into the SRS of RCS rats likewise delayed RD by preservation of retinal function [106]. It was initially demonstrated that rat BM-MSCs grafted into the SRS of RPE damaged rats, which were induced by sodium iodate, were able to proliferate and differentiate into retinal cells via expression of rhodopsin, glial fibrillary acidic protein and pan-cytokeratin [107]. Efforts also have been made to transplant cat BM-MSCs into the retina of felid species. The right eye of 24 cats after optic nerve injury accepted intravitreal injection of cat BM-MSCs, and it was found that MSCs could steadily express brain-derived neurotrophic factor but did not promote neural axon regeneration or differentiate into neuronal cells to mediate neuroprotection after traumatic optic neuropathy [108]. Moreover, following human BM-MSC transplantation, the degenerating retinal cells of RCS rats were also rescued without the requirement of immunosuppression [109].

#### Progress in the study of ADSCs

It was reported that human ADSCs have the capability to differentiate into neural retinal cells in vitro with paired box 6 protein (5a) gene expression [12]. In the laboratory of Li et al., subretinal transplantation of GFP-labelled human ADSCs into RCS rats effectively enhanced the survival rate of retinal cells, delayed RD and gave rise to increased visual function through the secretion of vascular endothelial growth factor, [110]. ADSCs are much more abundant and easier to harvest from donors with less invasive procedures, which makes them an important alternative to BM-MSCs [111]. Moreover, they expand faster, show more protein secretion (such as insulin-like growth factor-1, interferon-γ and basic fibroblast growth factor), and demonstrate a higher immunomodulatory capacity than BM-MSCs [111, 112]. According to the current literature, there is no significant difference between them in crucial mRNAs and protein expression (like Oct3/4 and Sox2) [113]. Collectively, these biological different data should be considered systematically when selecting ADSCs or BM-MSCs for specific clinical applications.

#### The main advantages and disadvantages of MSCs

Currently, MSC transplantation based on gene techniques is a better option in terms of neuroprotective effects, survival, integration and differentiation. Guan et al. explored erythropoietin gene-modified rat MSC therapy for sodium iodate-treated rats via SRS transplantation [114]. The improvements of retinal morphology and function were remarkable in two types of erythropoietin-rat MSCs relative to rat MSCs alone. Other typical examples included CX3CL1-expressing MSCs [115] and NT-4-engineered MSCs [116]. To date, most studies have supported the neuroprotective effect of MSCs [117], while rare evidence of neuronal replacement has been reported by means of MSC transplantation. According to observations by researchers, the mechanism of photoreceptor survival promotion is that MSCs strictly regulate their self-renewal capacity through anti-apoptotic, anti-inflammatory, immunomodulatory and angiogenic effects, which are based on MSC secretion of cytokines [118], growth factors and proteins, such as vascular endothelial growth factor, stromal cell-derived factor 1-alpha [119] and progranulin [12].

#### Clinical studies of MSCs

Clinically, following the feasibility of autologous BM-MSC transplantation demonstrated by Jonas et al. [120, 121], Siqueira et al. launched a Phase I trial (in 2009 at São Pauloin, NCT01068561) in patients with RP by injecting autologous BM-MSCs into the vitreous cavity [122, 123]. Then, a Phase II study (NCT01560715) was conducted based on some good results of preliminary clinical findings that the vision-related life quality of 20 patients improved statistically at three months post-treatment, whereas it deteriorated afterwards (by the 12th month) [17]. Another pioneering clinical trial (NCT01736059) led by Park (US) used intravitreal autologous BM CD34+ cells in six subjects (six eyes) with ischaemic disorder or RD. It seemed to be feasible and tolerated within six months of follow-up, yet beneficial effects need further exploration [124]. Improvement loss with time partly suggested that MSCs may not the best candidate for RD treatment. Most importantly, their dominant biological function is trophic support via a paracrine mechanism instead of cellular replacement. Therefore, the clinical application of hMSCs for replacement therapy in RD demands further investigation.

### Difficulties and prospects

RD commonly results from RPE and photoreceptor apoptosis. Much progress in stem/progenitor cell therapy for RD have been made through a succession of studies on RPCs, ESCs, iPSCs, and MSCs. They are currently regarded as promising therapeutic approaches for RD. However, one critical point is to choose the best stem/progenitor cell source for successful clinical application. Here, RPC populations are one of the most promising candidates because the manufacturing process is relatively simple, safe and straightforward. More importantly, RPCs originating from the developing retina have exhibited immune privilege as allografts so that better neuroprotection can be attained relative to other pluripotent cells [13]. Although the proliferation of donor RPCs is limited utilizing primary culture, RPCs can be viable beyond passage 20 by making full use of novel culture techniques. Compared with RPCs, the other three stem cell types have their own characteristics (Table 1). The ethical concerns are particular to clinical applications of hESCs involving the use of early human embryos. Regardless of the low risk of graft-host immune rejection, iPSCs can lead to tumourigenicity, mutations and epigenetic changes. Whether the extraneous four transcription factors induce reprogrammed iPSC abnormalities is still unclear [101]. MSCs predominantly protect retinal neurons from further dysfunction at early stages of RD rather than replace the lost and dead retinal cells at late stages. In addition, clinical trials are underway. The primary objectives of Phase I and II clinical trials are safety and efficacy, respectively. Both types of clinical trials require enough time and patient samples, although no major adverse event has been reported so far. At the same time, these successes of RD treatment will further represent a solid milestone for the treatment of other degenerative diseases in the brain and spinal cord in the near future because they all belong to central nervous system and share most common characteristics of the regenerative response.

## Conclusions

At present, although the efficacy and efficiency of stem/progenitor cell (excluding RPE)-based therapy for RD is generally restricted by the low rate of proliferation and/or differentiation in vitro and poor cellular survival, migration, integration and function in vivo, the therapeutics assisted by gene techniques, neuroprotective compounds, and artificial devices can be applied in RD treatment to fulfil clinical needs.

## Abbreviations

RD: retinal degeneration
AMD: age-related macular degeneration
RP: retinitis pigmentosa
RPE: retinal pigmented epithelium
RPCs: retinal progenitor cells
ESCs: embryonic stem cells
iPSCs: induced pluripotent stem cells
MSCs: mesenchymal stromal cells
BM-MSCs: bone marrow mesenchymal stromal cells
ADSCs: adipose-derived stromal cells
GFP: green fluorescent protein
ONL: outer nuclear layer
RA: retinoic acid
hRPCs: human retinal progenitor cells
SRS: subretinal space
RCS rats: Royal College of Surgeons rats
hESCs: human embryonic stem cells
SMD: Stargardt’s macular dystrophy
hiPSCs: human induced pluripotent stem cells
